# Case report: Remedial surgical treatment of aorto-duodenal fistula with infected aneurysm after endovascular aortic repair

**DOI:** 10.3389/fcvm.2022.975871

**Published:** 2022-10-11

**Authors:** Wen-Dong Li, Guang-Yan Wu, Bin Song, Jie Zhao, Xiao-Qiang Li, Min Zhou

**Affiliations:** Department of Vascular Surgery, The Affiliated Drum Tower Hospital of Nanjing University Medical School, Nanjing, China

**Keywords:** aorto-duodenal fistula, infected aneurysm, endovascular aortic repair complications, aortic abdominal aneurysm, ruptured iliac artery aneurysm

## Abstract

Aorto-duodenal fistula (ADF) is a rare cause of upper gastrointestinal bleeding, but it is associated with high mortality. It usually occurs in patients with prior aortic surgery or who have undergone aortic graft placement. Abdominal aortic aneurysm (AAA) might be a cause of primary ADF, which could develop into sudden shock. Because ADF is difficult to diagnose, surgery to correct it has a poor outcome. We here report the successful treatment of an ADF complicated with infected AAA after endovascular repair of a ruptured aneurysm of the iliac artery.

## Case report

**FIGURE 1:**
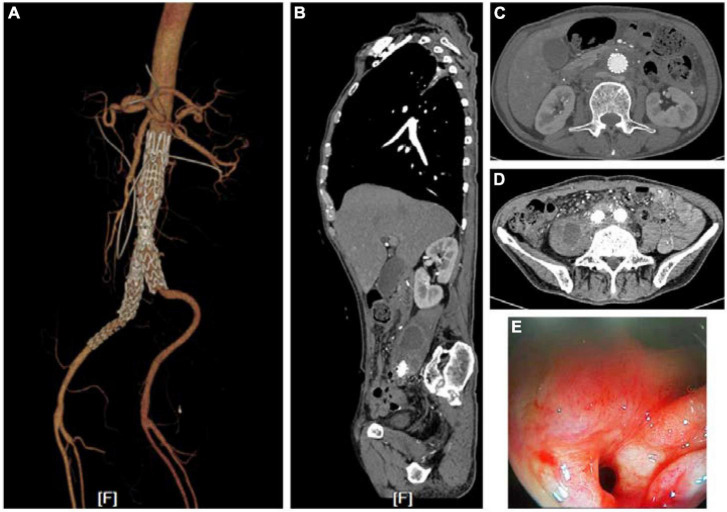
Images of CTA and endoscopy before treatments. **(A)** Endovascular repair and drainage had been performed when the patient was hospitalized. **(B–D)** A low-density shadow hinting at an abscess in the right psoas major muscle and around the stenting. **(E)** Fistulae in the horizontal part of duodenum found via endoscopy.

A 71-year-old man underwent two rounds of endovascular repair treatment for rupture of an aneurysm of the right iliac artery 6 months prior to the index operation. He presented with recurrent hematemesis, melena, and intermittent fever. He was treated with antibiotics according to the findings of the bacteriology examination of blood samples. PET-CT suggested a gastrointestinal stromal tumor surrounding the abdomen. Emergency endoscopy revealed a fistula of duodenum and minor hemorrhage. The fistula was found in the horizontal part of duodenum. Computed tomography angiography (CTA) scan had revealed an infected abdominal aortic aneurysm (iAAA) with low-density image surrounding the AAA and the right iliac artery (Figure 1). However, there was no evidence of contrast leakage into the intestinal tract. Blood tests at the time of the patient’s arrival revealed severe anemia, renal dysfunction, elevated white blood cell count, and high levels of serum procalcitonin and C-reaction protein. Blood culture found *Escherichia coli*, which was sensitive to vancomycin. Based on these findings, aorto-duodenal fistula (ADF) and iAAA were suspected.

**FIGURE 2:**
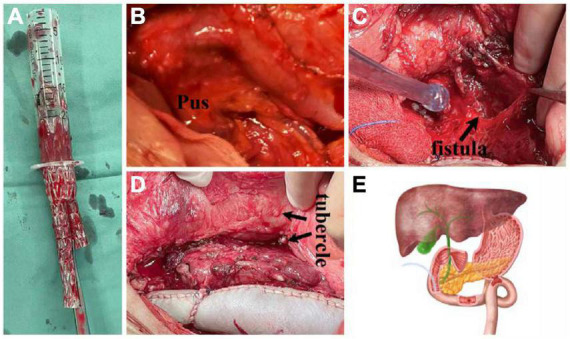
Images taken during the operation. **(A)** The stent in the abdominal artery was removed using an injector. **(B,C)** Pus and fistula were found surrounding the horizontal part of duodenum. **(D)** The infected abdominal aortic artery was replaced with a bovine pericardium biological mesh. There were many small masses around the abdomen. Pathological analysis showed this mass to be Schistosoma eggs. **(E)** A schematic diagram of the surgery in the horizontal part of duodenum.

After a week of antibiotic therapy with vancomycin (500 mg), the fever was controlled. Then, a series of surgeries were performed. The stents in the aneurysm were removed using an injector and supra (Figure 2) or infrarenal aortic cross-clamping technique. First, the artery was clamped at the suprarenal aortic site. The primary purpose of this was to reduce blood loss, but it also rendered the endograft easier to remove. After the graft was removed, we clamped the infrarenal aortic site to restore the blood flow to the kidney and prevent renal failure. The aortic aneurysm was replaced *in situ* with an artificial blood vessel made in-house using bovine pericardium biological mesh. The distal end of this artificial blood vessel was sutured to the left common iliac artery. The right common iliac artery and right internal iliac artery were surrounded with pus, which was removed carefully. The stumps of the two arteries were ligated. Femoral artery bypass grafting was performed to restore circulation in the right lower extremity.

There were two 3-mm-wide fistulas between the anterior wall of the AAA and the horizontal part of the duodenum. This was consistent with the findings of endoscopy. The perforated region of the duodenum was resected. A gastrojejunal Roux anastomosis was performed. The cavity was irrigated with 10 L of saline. The momentum was placed between the aorta and duodenum. Many scattered nodules were found around the abdomen. The results of the histological examination showed that chronic inflammatory cells had focally infiltrated into the aneurysmal wall, and that the nodules, which PET-CT suggested could be gastrointestinal stromal tumors, were verified as Schistosoma eggs. Bacterial culture of the pus in the aneurysm showed *Escherichia coli* infection, as in the blood culture before surgery. Antibiotic therapy was maintained postoperatively for at least 6 months with no evidence of recurrent infection as of 6 months into follow-up. There was no blockage of the aorta and femoral artery bypass graftings at CTA 3 months after the indexed operation.

## Discussion

Aorto-duodenal fistula is the most common aorto-enteric fistula (1). It most commonly originates from an atherosclerotic AAA (2). It usually involves at the third or fourth duodenal segment, presenting with upper gastrointestinal bleeding but not obstructive syndrome. This may be primary caused by spontaneous communication between the lumen of aortic aneurysm and the intestinal loop, or secondarily caused by surgical repair of aneurysms, becoming detectable months or even years after surgery (1, 3). ADF should be considered for patients with upper gastrointestinal bleeding and a history of surgery with artificial blood vessels or stents in the aorta. Endoscopy combined with CT angiography or arteriography could confirm a definitive diagnosis (4). Delay in diagnosis and treatment has been historically associated with extremely high mortality (5).

**FIGURE 3:**
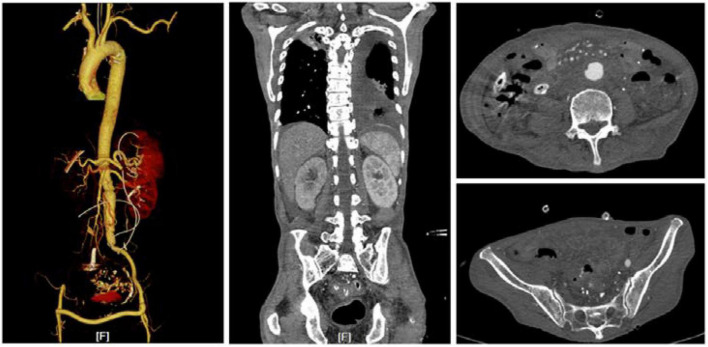
CTA result at 1 month after the operation. Abscesses in the abdomen were cured. The bypass vessels and the lumen were unobstructed.

Aorto-duodenal fistula management goals include maintaining distal perfusion after controlling the hemorrhage and preventing recurrent infection (6). Once ADF is diagnosed, preventive measures, such as antibiotic therapy, delicate surgery for eradication of septic focus with thorough debridement of infected and devitalized tissue, and reconstruction of the excised aorta by extra-anatomic or an *in situ* route, are required (7, 8). For patients with hemodynamic instability, EVAR can serve as a bridging therapy in cases with problematic bleeding. It can seal the fistula and stop bleeding rapidly (9, 10). For patients with severe purulent infection in AAA and periaortic tissue, extra-anatomic bypass was suggested to be an alternative approach for treatment (11). However, results have shown this method to have a high mortality rate because of high risk of limb loss and aortic stump blowout. *In situ* reconstruction could be performed by homograft, antibiotic-impregnated prosthetic graft, or autologous femoral vein graft. The results of this treatment might depend on the patient’s condition, the extent of vascular disease, and the virulence of the bacteria (8, 12, 13). In this case, the infected aortic artery was replaced *in situ* with an artificial blood vessel made by bovine pericardium biological mesh. It also produced good results in the reconstruction of the infected aorta. For the duodenal fistula and infection in the abdomen, gastrojejunal Roux anastomosis was performed. This treatment also produced good results in the repair of the duodenal fistula (Figures 2, 3).

## Conclusion

We here reported a case of ADF complicated with iAAA. Diagnosis was made via CTA and endoscopy. Explorative laparotomy, including removal of stents and AAA placement of an artificial blood vessel made of bovine pericardium with biological mesh, grafting of the femoral artery bypass, removal of pus from the right iliac artery, resection of the perforated region of the duodenum, and gastrojejunal Roux anastomosis, was performed with excellent results.

## Data availability statement

The original contributions presented in this study are included in the article/supplementary material, further inquiries can be directed to the corresponding authors.

## Ethics statement

Written informed consent was obtained from the participant(s) for the publication of this case report. Written informed consent was obtained from the individual(s) for the publication of any potentially identifiable images or data included in this article.

## Author contributions

W-DL: manuscript writing. X-QL and MZ: final approval of the manuscript. All authors participate in the treatments of this case.
